# Diseases of the Human Body Which Have Been Traced to the Action of Mouth-Bacteria

**Published:** 1891-11

**Authors:** W. D. Miller

**Affiliations:** Berlin.


ARTICLE III.

### DISEASES OF THE HUMAN BODY WHICH HAVE BEEN TRACED TO THE ACTION OF MOUTH-BACTERIA.

BY W. D. MILLER, M. D., PH. D., BERLIN.

Extracts from a paper on "The Human Mouth as a Focus of Infection," published in the September number of the *Dental Cosmos*.

1. *Decay of the Teeth.*—In conformity with the nearly unanimous verdict of all recent investigations, decay of the teeth must be set down as the most widespread of all parasitic diseases to which the human body is subject; and although, as far as the life of the patient is concerned, the prognosis is exceedingly good, and decay of the teeth may be pronounced one of the most trivial disturbances in the human economy, yet, if we take into consideration the results which follow a case of general decay, particularly in the mouth of young or weak persons, it often becomes a disease of very grave nature.

I venture to say that most practitioners of dentistry will agree with me that the havoc wrought by dental caries in the mouths of vast numbers of children, or even adults, among the lower classes, is a much more serious thing than an attack of chicken-pox, rubeola, or even measles.

2-4. *Pulpitis, Gangrene of the Pulp, Pericementitis.*—Inflammation of the dental pulp, with the exception of the comparatively few cases where it is the result of trauma or of calcareous formations in the pulp-chamber, erosion, abrasion, etc, is due directly or indirectly to the parasitic influences, while gangrene of the pulp can never have any other origin under any circumstances.

Pericementitis apicalis, the form of pericementitis which is most severe and gives rise to the most serious consequences, is likewise of parasitic origin, being produced by germs or their products, or by both together passing from the root-canal through the apical foramen.

5. *Alveolar Abscess.*—Alveolar abscess is an infectious disease, primarily of a local character, but frequently, or usually, accompanied by general symptoms of varying intensity, and sometimes attended by complications of a most serious nature. Severe cases of alveolar abscess, particularly in weak persons, not unfrequently present symptoms of an alarming nature. * * * I wish to call particular attention to the many cases in which it has terminated fatally through the supervention of septicæmia or pyæmia.

It must be constantly borne in mind that wherever micro-organisms are accumulated in large masses in any part of the body, the possibility of their being carried to other parts through the blood or lymph-channels, and of their producing, accordingly, metastatic abscesses wherever a point of diminished resistance exists, can never with certainty be excluded. * * * In like manner, general blood-poisoning (septicœmia), with speedily fatal termination, has been seen to result from accumulations of infectious material about the roots of a tooth.

6, 7. *Ostitis, Osteomyelitis.*—Every severe inflammation of the pericementum is naturally accompanied by more or less inflammation of the bone-marrow, or of the bone (ostetis), or of both together (osteomyelitis).

8, 9. *Periostitis and Necrosis.*—A slight inflammation of the periosteum of the alveolar process and a slight necrosis of the bone necessarily accompany all abscesses in which the pus makes its way to the surface of the bone. * * * Not unfrequently, however, periosteal inflammations resulting from caries are of an exceedingly violent character, with intense, continuous pain, often lasting for days, enormous swelling, debility, fever, chills, sometimes terminating fatally. * * * Necrosis is but a more advanced stage of osteomyelitis and periostitis. The bone, deprived of all sources of nutrition, dies (becomes necrotic), and is afterwards thrown off by the surrounding tissues in the form of a so-called sequestram.

10. *Dental Fistulæ.*—In this connection I refer in particular to those fistulæ of dental origin which open on the neck, shoulder, arm, or breast, thus giving rise to so-called "running sores," which of course defy all treatment until the true source is discovered.

11. *Septicæmia.*— Many cases may be found in medical and dental literature, in which a general infection of the blood causing the death of the patient in a few hours has resulted from the accumulation of pus about a diseased tooth, or from operations in the mouth.

12. *Pyæmia.*—Chronic pyæmia presents itself in form of abscesses of varying intensity occurring in different parts of the body, healing spontaneously at one point, only to break out again at some other more or less remote. An abscess at the point of the finger or on the toe may originate in a diseased tooth as well as an abscess at the point of the root.

13. *Meningitis, Encephalitis, Abscess of the Brain, etc.*—A superficial examination of the relations of the teeth to the cavity of the skull, will show us that an inflammatory process incited by the teeth of the upper jaw may reach the brain cavity either through the maxillary sinus, nasal cavity, and cribriform plate of the ethmoid bone (or directly through the nasal cavity and ethmoid), or through the pterygoid fossa and foramina at the base of the skull, or by way of the spheno-maxillary fossa, inferior sphenoidal fissure, orbit, etc. Inflammatory processes in the lower jaw ascending the ramus usually obtain entrance to the skull cavity by way of the orbit, less frequently, it seems, through the pterygoid fossa.

14. *Impeded Eruption of Wisdom Teeth.*—The chronic state of irritation upon the gums and periosteum resulting from impacted wisdom-teeth, and the consequent state of diminished resistance, make it possible for micro-organisms, which obtain entrance between the crown of the tooth and the overlapping gums (assisted as they so frequently are by the irritating action of small particles of food undergoing fermentation), to multiply in large numbers and, penetrating along the course of the distal root into the depths of the jaw, to bring about the series of disturbances, ostitis, osteomyelitis, periostitis, phlegmon, trismus, and in some cases necrosis or even septicæmia.

15. *Pyorrhœa Alveolaris.*—There are many reasons for believing that pyorrhœa alveolaris has an origin similar to that of the suppurative processes associated with the impeded eruption of the lower wisdom-teeth. * * * The evil results of allowing this disease to gain the upper hand manifest themselves not only in the impairing or complete loss of the efficiency of the teeth as organs of mastication, but also, as has been expressed by Galippe, when a secretion of matter in the mouth becomes general, patients may suffer from fever, loss of appetite, stiffness, severe disturbances of the alimentary canal, insomnia, subictoritic discoloration of the skin, etc.

16. *Disturbances in the Alimentary Tract.*—The mouth, as has been sufficiently well established, furnished one of the chief sources for the constant recruiting of the bacteria of the stomach and intestines. Not only this, but the constant swallowing of decomposing matter and of pus from an improperly cared for mouth may lead to the most serious disturbances, both acute and chronic.

17.—*Diseases of the Lungs—Croupous Pneumonia.*—The uniform results obtained by investigators on the subject of pneumonia for the last five years leave little room for doubt that the cause of this important disease is to be sought for in a species or group of micro-organisms which are constantly present in the sputum of persons suffering from pneumonia and very frequently even in the saliva of quite healthy people. * * * Furthermore, the micrococcus of pneumonia not only does not proliferate at the ordinary temperature of the air, but, what is of still greater importance, soon loses its virulence when cultivated out of the body even under the most favorable conditions, which is still another potent reason for the supposition that in pneumonia the mouth and not the air is the direct source of the infection.

18. *Infiltration of the Surrounding Tissue and Chronic Swelling of the Lymphatic Glands in the Region of the Lower Jaw and Neck.*—The casual relation of a diseased condition of the teeth in this affection has been clearly enough established by Odenthal, who found glandular swellings in ninety-nine per cent. of all children who suffered from badly decayed teeth, and only in forty-nine per cent. of those with sound teeth.

19. *The Infectious Anginæ* (*Tonsillitis*, *Amygdalitis Infectiosa*, *etc.*)—It is now commonly recognized that the tonsils may harbor various pathogenic bacteria in their lacunæ without any appreciable evil consequences, until, through some cause or other, which may be of a very trivial nature, their action manifests itself either in form of a local or general infection. Particularly tonsils which are chronically inflamed, hypertrophied, are dangerous accumulators of pathogenic germs.

20. *Augina Ludovici.*—Sufficient evidence has been accumulated to render it highly probable that this severe, though rare affectation, is the result of the invasion o micro-organisms through slight wounds, ulcerations, or other breaks in the continuity of the mucous membrane, or by way of diseased teeth, or of the tonsils, or of the ducts of the sublingual and submaxillary glands.

21. *Diseases of the Maxillary Sinus.*—These are of such frequent occurrence that every practitioner must have seen one or more cases. It is not necessary to refer to that fact that they are in the vast majority of cases the result of the action of mouth-bacteria.

22. *Pneumococcus Abscesses.*—It has been well established that the so-called pneumococcus possesses invasive properties of the highest order, so that there is hardly any part or organ of the human body which may not fall a prey to its action. * * * In such cases the coccus is transported from the mouth to other parts of the body through the blood or lymphatics, or as is often the case in otitis, meningitis, etc., by a direct spreading of the affection from the mouth to the neighboring cavities.

23. *Disturbances Resulting from the Absorption of Products of Putrefaction Through the Mucous Membrane of the Mouth.*—In persons of uncleanly habits, who neglect the care of the mouth, and especially who allow rubber plates to remain in the oral cavity for weeks together, constantly covered with a thick coating of putrefying mucus and food, loss of appetite, nausea, vomiting, and chronic indigestion may result from the prolonged action of the products of decomposition upon the mucous membrane of the mouth and pharynx.

24. *Stomatitis Ulcerosa* (*S. Scorbutica, S. Mercurialis.*)—These are nothing more or less than the result of the invasion of pyogenic and saprophytic bacteria of the mouth upon a tissue which has already suffered a severe diminution in its powers of resistance through the general primary affection.

25. *Actinomycosis.*—This disease is of so frequent occurrence and its connection with the mouth so apparent, that it requires only to be mentioned to carry with it an argument for a more commensurate estimation of the importance of careful attention to the hygiene of the mouth.

26. *Noma.*—Although of comparatively rare occurrence, this disease excites particular interest on account of the fearful ravages which it produces and the rapidity with which it advances, so that in the space of three or four days the whole cheek, nose, eyelids, mucous membrane of the jaw and soft palate may be transformed into a necrosed, putrefying mass.

27. *Pharyngomycosis* (*Mycosis Tonsillaris Benigna.*)—An infection caused by a proliferation of saprophytic bacteria in the lacunæ of the tonsils.

28. *Stomatomycosis.*—This is caused by the colonization of sarcina on the mucous membrane of the cheeks.

29. *Thrush.*—A well known disease caused by the invasion of a yeast-fungus, Saccharomyces albicans. These are all troubles of undoubted parasitic nature.

33. *Inflammation and Suppuration.*—These affections of the salivary glands, in particular of the parotid, must also be mentioned as troubles which in many cases owe their origin to mouth-bacteria which find their way through the ducts to the body of the gland.

34. *Diphtheria.*—The fact that an attack of diphtheria may be provoked by slight wounds in the mouth, or by the presence of diseased teeth, and the fact that the extirpation of the tonsils has proved to be one of the most successful prophylactic measures against diphtheria, seem to point to the conclusion that the human mouth and throat harbor the diphtheritic bacilli under normal conditions until the proper moment arises for them to assert their specific action.

35. *Tuberculosis.*—Many cases are on record in which primary tuberculosis of the mouth has made its first appearance around diseased teeth, or roots of teeth or following extractions and other operations in the mouth.

36. *Syphilis.*—The question of syphilitic infection through dental operations (extraction, filling,) through unclean rubber-dam, instruments, drinking-glasses, through kisses, transplantation of teeth, bites, etc., has been so frequently discussed in dental periodicals, and must be so familiar, that a simple statistical inquiry into the question will suffice to show the importance of exceeding great care on the part of the dentist to prevent transmitting the disease to innocent patients.

37. *Infections Following Operations in the Mouth.*—In recent years the demand for the adoption by dental surgeons of the same antiseptic measures observed by the general surgeon has constantly become more and more imperative. Attention has been repeatedly called to the fact that bloody operations in the mouth, such as tooth-extractions, performed, as too many of them are, without the slightest regard to the principle of asepsis, often lead to infections of serious nature, which might have been easily avoided; not only that, but carelessness in regard to cleansing the instruments after every operation frequently results in the communication of disease from one individual to another.

38. *Infections Resulting from Wounds with Dental Instruments.*—Numerous cases have recently been brought to light in which slight wounds upon the hand, inflicted by instruments used in operations upon the teeth, also scratches of the fingers on sharp roots, have resulted in infections of a most serious nature.

39. *Stomatitis Epidemica—Foot and Mouth Disease in Man.*—Under the above title, I wish to refer to an infection, which, as will be shown, in all probability, has the same origin as the foot and mouth disease in cattle, but which being communica ed directly from man to man, becomes more virulent than when communicated from cow to man, the relation presumably being the same as that between variola and varioloid.

The epidemic referred to occurred in one of the suburbs of Berlin, a district having a population of nine thousand persons, of which over six thousand have suffered from the disease in the last eighteen months.

---

ARTICLE IV.

### THE CARE OF CHILDREN'S TEETH.

